# Supplementary material for: Mapping the evolution of fertility support policies in China: A content and instrumental analysis
Source: PLoS One. 2025 Oct 9;20(10):e0332137. doi: 10.1371/journal.pone.0332137 (PMC12510515; doi:10.1371/journal.pone.0332137)
Supplement: S1 Appendix — (ZIP) [file pone.0332137.s001.zip › S1 Appendix. 226 original policy documents/47-国家卫生健康委员会关于印发母婴安全行动计划(2018―2020年)和健康儿童行动计划(2018―2020年)的通知(FBM-CLI-4-322501).doc]

国家卫生健康委员会关于印发母婴安全行动计划(2018―2020年)和健康儿童行动计划(2018―2020年)的通知

**发布部门：** [国家卫生健康委员会](https://www.pkulaw.com/law/chl?Aggs.IssueDepartment=60321) [机构沿革](javascript:void(0))

**发文字号：**国卫妇幼发〔2018〕9号

**发布日期：**2018.04.27

**实施日期：**2018.04.27

**时效性：** [现行有效](https://www.pkulaw.com/law/chl?Aggs.TimelinessDic=01)

**效力级别：** [部门工作文件](https://www.pkulaw.com/law/chl?Aggs.EffectivenessDic=XE0303)

**法规类别：** [卫生综合规定](https://www.pkulaw.com/law/chl?Aggs.Category=09601)

国家卫生健康委员会关于印发母婴安全行动计划（2018-2020年）和健康儿童行动计划（2018-2020年）的通知
（国卫妇幼发〔2018〕9号）

各省、自治区、直辖市及新疆生产建设兵团卫生计生委：
　　为学习贯彻党的十九大精神，落实[“健康中国2030”规划纲要](https://www.pkulaw.com/chl/a55bb2910f6162efbdfb.html)，切实保障母婴安全，促进儿童健康成长，我委制定了《母婴安全行动计划（2018-2020年）》和《健康儿童行动计划（2018-2020年）》（可从国家卫生健康委员会网站下载）。现印发给你们，请认真组织实施，确保工作取得实效。
　　附件：1. 母婴安全行动计划（2018-2020年）
　　2. 健康儿童行动计划（2018-2020年）

国家卫生健康委员会
2018年4月27日

　　附件1

　　母婴安全行动计划（2018-2020年）

　　为提高妇幼健康服务质量和水平，切实保障母婴安全，维护妇女儿童健康权益，自2018年起，在全国组织实施母婴安全行动计划。
　　**一、**总体要求
　　以预防和减少孕产妇和婴儿死亡为核心，以落实母婴安全保障制度为重点，以提升医疗机构服务能力为抓手，以强化质量安全管理为保障，为群众提供安全、有效、便捷、温馨的妇幼健康服务，全力维护妇女儿童健康。

　　**二、**行动目标
　　自2018至2020年，通过开展母婴安全行动，提升妇幼健康服务水平，降低孕产妇死亡率和婴儿死亡率，到2020年全国孕产妇死亡率下降到18/10万，全国婴儿死亡率下降到7.5‰。在此基础上，树立一批母婴安全示范单位。

　　**三、**行动范围
　　开展助产技术服务的医疗机构（以下简称助产机构），重点是二级及以上综合性医院、妇幼保健院和妇产医院。

　　**四、**行动内容
　　（一）妊娠风险防范行动。
　　1.提升风险防范意识。以科学备孕、孕产期保健、安全分娩为重点，制订孕产妇健康教育工作计划，开发针对性的健康教育材料。利用电视、广播、报刊、网站、微信、微博等大众媒体，制作参与科普节目，积极撰写科普文章，广泛开展健康教育与健康促进，普及孕育健康知识，提升群众健康素养，使每个孕产妇成为自身健康第一责任人，积极参与配合医疗保健活动，提升自我保健和风险防范意识。
　　2.做好备孕咨询指导。将妊娠风险教育作为孕妇学校开班第一课。设立生育服务咨询室，汇集妇科、产科、生殖、遗传、心理、中医等专业力量开展多学科协作诊疗，规范提供生育力评估和备孕指导。综合评估妇女基础健康状况、生育能力和年龄等因素，客观告知妊娠几率和风险，引导群众正确认识高龄高危妊娠风险。
　　3.开展妊娠风险评估。按照《妊娠风险评估与管理工作规范》要求，开展孕产妇妊娠风险筛查和评估，对孕产妇进行分级分类管理，在《母子健康手册》及相应信息系统作出明显标注。对于妊娠风险分级为“黄色（一般风险）”、“橙色（较高风险）”、“红色（高风险）”和“紫色（传染病）”的孕产妇，应当建议其在二级以上医疗机构接受孕产期保健服务和住院分娩。对患有疾病可能危及生命不宜继续妊娠的孕产妇，由副主任以上任职资格的医师进行评估和确诊，告知继续妊娠风险，提出科学严谨的医学建议。
　　4.提供生育全程服务。使用全国统一的《母子健康手册》，整合孕前保健、孕期保健、住院分娩、产后避孕、儿童保健等内容，提供系统、规范的优生优育全程服务，打造无缝连接“一条龙”服务链。规范有序开展产前筛查与产前诊断服务，严格落实预防艾滋病、梅毒和乙肝母婴传播等综合防控措施。指导产妇分娩后及时采取避孕措施，减少非意愿妊娠，合理控制生育间隔。
　　（二）危急重症救治行动。
　　5.加强高危人群管理。筛查妊娠危险因素，识别高危孕产妇，对妊娠风险分级为“橙色”、“红色”和“紫色”的孕产妇严格实行高危孕产妇专案管理，保证专人专案、全程管理、动态监管、集中救治，确保做到“发现一例、登记一例、报告一例、管理一例、救治一例”。密切监测、治疗妊娠合并症和并发症，根据病情需要及时转诊。
　　6.查找救治薄弱环节。每半年组织产科、儿科、重症医学等业务科室和医务科等职能部门召开至少1次联席会议，梳理在危重孕产妇和危重新生儿救治方面存在的管理、技术问题，完善诊疗预案和管理制度，建立孕产妇用血、转运等保障机制。积极探索开展孕产妇危重症评审。
　　7.针对死因完善预案。结合区域孕产妇和新生儿死亡评审情况以及本机构实际，针对产后出血、新生儿窒息等主要死因建立抢救流程与规范，明确相关科室和人员职责任务，确保急救设备和药品随时处于功能状态。建立完善危重孕产妇和新生儿救治医患沟通和媒体沟通机制。
　　8.建立救治协调机制。设立产科安全管理办公室，由分管院长具体负责，协调建立危重孕产妇和新生儿救治、会诊、转诊等机制。建立院内多学科危重孕产妇和新生儿急救小组。完善产科、儿科协作机制，鼓励产科与儿科共同确定分娩时机，儿科医师按照院内会诊时限要求准时到达。
　　（三）质量安全提升行动。
　　9.完善质量管理体系。落实《[医疗质量管理办法](https://www.pkulaw.com/chl/8632af233065e26cbdfb.html)》，成立医疗质量管理专门部门，负责本机构的医疗质量管理工作。产科、儿科应成立本科室医疗质量管理工作小组，组长由科室主要负责人担任，指定专人负责日常质量管理工作。每年开展不少于1次医疗质量安全案例警示教育。
　　10.加强医疗安全管理。严格遵守医疗质量安全核心制度。强化产科探视管理，新生儿在院期间佩戴身份识别腕带，完善新生儿出入管理制度和交接流程，做到身份有识别、交接有登记。规范处理医疗废物，依法依规妥善处理胎盘和死胎（死婴）。加强新生儿病房、临床检验实验室、人类辅助生殖技术实验室等重点部位医院感染管理，严格执行消毒隔离、手卫生和医院感染监测等规定。建立风险监测、预警以及多部门协同干预机制，严格执行医院感染暴发报告制度，有效防范医院感染。
　　11.促进质量持续改进。针对手术室、产房、新生儿病房等重点部门，围绕关键环节和薄弱环节制定质量评价标准，建立相应的质量安全考核指标。科室质量管理小组应当每月开展自我评估与分析，制订并落实质量持续改进措施。运用质量管理工具分析医疗质量安全信息。
　　12.定期报送母婴安全信息。按照卫生计生行政部门要求，定期报送住院分娩、孕产妇死亡、新生儿死亡、出生缺陷等个案数据以及服务资源数据，发生孕产妇死亡后第一时间通报辖区县级妇幼保健机构。建立医疗质量（安全）不良事件信息采集、记录和报告相关制度。
　　（四）专科能力建设行动。
　　13.加强临床专科建设。以各层级临床重点专科建设为抓手，提升产科、儿科专科诊疗水平。综合性医院着力加强妊娠合并症处置、危重孕产妇多学科联合救治，重点提升疑难重症诊疗能力。分娩量较大的妇幼保健院、妇产医院着力加强产科亚专科和新生儿科建设，逐步建立产科重点专病医疗组。积极推广中医适宜技术和方法，开展中成药合理使用培训，促进孕产妇和婴幼儿安全应用中药。加强危急重症中西医临床协作，提升诊疗救治能力。
　　14.促进保健专科发展。开展妇幼保健专科示范单位建设，丰富服务内涵，拓展服务内容，加强人才建设，促进预防保健与临床医疗融合发展，不断提升孕前保健、孕产期保健、产后保健、儿童眼保健、儿童听力保健等保健专科服务能力和水平。发挥中医治未病优势，推广应用中医防病保健方法，扩大中医药在孕育调养、产后保健等方面的作用。
　　15.强化专业技能培训。结合辖区孕产妇和新生儿死亡评审情况，确定技能培训主题。每季度开展不少于1次专项技能培训和快速反应团队急救演练，提升快速反应和处置能力，紧急剖宫产自决定手术至胎儿娩出时间（DDI）应当努力控制在30分钟以内并逐步缩短。保障产科医师、助产士、新生儿科医师每年至少参加1次针对性继续医学教育。
　　16.推进科研攻关和临床转化。以临床应用为导向，在产后出血、前置胎盘、胎盘植入等妊娠合并症和并发症处理及出生缺陷防治、早产风险提示、早产儿救治等方面组织开展科研攻关。加快推进产后出血防治、新生儿复苏等适宜技术的推广应用。
　　（五）便民优质服务行动。
　　17.优化诊疗资源配置。按照开放床位和配置标准，足额配备医护人员和设施设备，确保服务资源与服务量相匹配。在门诊合理安排B超等设备，增加胎心监护等可穿戴设备，逐步缩短检查等候时间。优化产科诊室布局和服务流程，集中产科门诊、超声检查、胎心监护、采血、尿检、缴费等环节，努力提供“一站式”服务。完善自助服务设备，提供便民服务设施，在儿科和儿童保健门诊设立母乳喂养室。
　　18.推进全面预约诊疗。三级综合医院和三级妇幼保健院的产科预约诊疗率≥70%，产前检查复诊预约率≥90%。在保障危重孕产妇救治的前提下，推广预约住院分娩，对预约孕产妇优先安排住院床位。
　　19.提供便民利民服务。通过诊间结算、移动支付等方式，减少患者排队次数，缩短挂号、缴费、取药排队等候时间。提供自助打印、网络查询、手机信息等多种形式的检查检验结果查询服务。规范落实孕前优生健康检查、产前筛查、新生儿疾病筛查等惠民利民政策和措施。
　　20.倡导温馨舒适分娩。营造温馨、舒适的产房环境，提供以产妇为中心的人性化分娩服务。积极开展专业陪伴分娩等非药物镇痛服务，鼓励开展药物分娩镇痛服务，有条件的医疗机构可开展家属陪伴分娩。

　　**五、**行动步骤
　　（一）2018年工作安排。
　　1.2018年1-5月，国家卫生健康委员会印发《母婴安全行动计划》部署相关工作。各省级卫生计生行政部门根据行动计划和当地实际，制订本省（区、市）具体实施方案和年度工作计划，落实各项工作措施，做好动员部署、宣传发动工作，调动医疗机构和医务人员积极性。各省（区、市）的实施方案要对孕产妇死亡率和婴儿死亡率较高的边远地区、贫困地区和少数民族地区等重点区域制订针对性的策略措施。
　　2.2018年6-12月，医疗机构落实母婴安全行动计划。国家卫生健康委员会组织督导检查，推动落实母婴安全行动计划。国家卫生健康委员会和省级卫生计生行政部门组织发现先进典型，挖掘一批母婴安全示范单位。国家卫生健康委员会和省级卫生计生行政部门组织对示范单位进行宣传报道。
　　（二）2019年工作安排。
　　1.2019年1月，总结2018年度行动计划落实情况。
　　2.2019年2月，国家卫生健康委员会通报2018年度情况，部署2019年工作。对2018年度母婴安全示范单位进行表扬，在全国会议上进行经验交流。
　　3.2019年3-12月，医疗机构落实母婴安全行动计划。国家卫生健康委员会组织督导检查，推动落实母婴安全行动计划。国家卫生健康委员会和省级卫生计生行政部门组织发现先进典型，挖掘一批母婴安全示范单位。国家卫生健康委员会和省级卫生计生行政部门组织对示范单位进行宣传报道。
　　（三）2020年工作安排。
　　1.2020年1月，总结2019年度行动计划落实情况。
　　2.2020年2月，国家卫生健康委员会通报2019年度情况，部署2020年工作。对2019年度母婴安全示范单位进行表扬，在全国会议上进行经验交流。
　　3.2020年3-12月，医疗机构落实母婴安全行动计划。国家卫生健康委员会组织督导检查，推动落实母婴安全行动计划。国家卫生健康委员会和省级卫生计生行政部门组织发现先进典型，挖掘一批母婴安全示范单位。国家卫生健康委员会和省级卫生计生行政部门组织对示范单位进行宣传报道。
　　4.2020年10-12月，国家卫生健康委员会对母婴安全行动计划3年实施情况进行总结，推进母婴安全工作常态化制度化。

　　**六、**行动要求
　　（一）细化落实行动措施。地方各级卫生计生行政部门要根据当地实际，制订具体实施方案，明确时间表、路线图，夯实工作责任，形成“一级抓一级、层层抓落实”的工作局面。国家卫生健康委员会将确定部分重点联系医院，强化督促指导，定期了解工作进展。各级卫生计生行政部门也要建立重点联系单位制度，督促医疗机构采取措施切实落实母婴安全行动计划。
　　（二）发挥典型示范作用。国家卫生健康委员会委属委管医院要发挥“国家队”表率作用，省级综合医院、妇幼保健院要发挥区域“龙头”作用。地方各级卫生计生行政部门要深入挖掘、树立先进典型，通过示范引领，带动辖区各级各类医疗机构落实母婴安全行动计划。国家卫生健康委员会将根据各地推荐的示范单位，遴选出一批全国示范单位组织系列报道，供各地交流学习。
　　（三）加强舆论宣传引导。地方各级卫生计生行政部门要加强母婴安全行动宣传，及时介绍工作进展，宣传典型机构、人员和事例，营造良好舆论范围。积极宣传扎根基层、情系群众的一线医护人员，增强医护人员职业荣誉感。
　　各地要将保障母婴安全摆在卫生健康工作的突出位置，精心组织实施，确保各项措施全面落实到位。各省（区、市）要将工作进展情况、做法经验等及时向我委报告。
　　附件2

　　健康儿童行动计划（2018-2020年）

　　儿童健康是全民健康的基础，是经济社会可持续发展的重要保障。为进一步提高儿童健康水平，依据《[中华人民共和国母婴保健法](https://www.pkulaw.com/chl/34423ea29b054d35bdfb.html)》、《[“健康中国2030”规划纲要](https://www.pkulaw.com/chl/a55bb2910f6162efbdfb.html)》、《中国儿童发展纲要（2011-2020年）》，制定本行动计划。

　　**一、**基本原则
　　坚持儿童优先，全面发展。在公共政策制定实施过程中，实行儿童优先发展战略，关注儿童健康事业，促进儿童体格、生理、心理、社会适应能力全面发展，为经济社会可持续发展提供健康人力资源。
　　坚持预防为主，防治结合。普及儿童健康知识，推行儿童健康生活方式，加强儿童健康管理，落实早发现、早诊断、早治疗的疾病防控策略，降低儿童疾病负担，促进儿童健康成长。
　　坚持问题导向，共建共享。围绕儿童健康突出问题和薄弱环节，动员全社会力量，采取针对性措施，保障儿童健康，促进家庭幸福、社会和谐。
　　坚持统筹协调，均衡发展。以基层为重点，加大对农村和贫困地区儿童健康事业发展的投入，补齐短板，夯实基础，缩小城乡、地区之间差距。
　　坚持道路自信，创新驱动。坚持保健与临床相结合、个体与群体相结合、中西医相结合，借鉴国内外先进经验，因地制宜，勇于创新，走出具有中国特色的儿童健康事业发展道路。

　　**二、**主要目标
　　到2020年，覆盖城乡的儿童健康服务体系进一步完善，儿童医疗保健服务能力不断提升，儿童健康水平得到提高。实现以下具体目标：
　　--婴儿死亡率和5岁以下儿童死亡率分别控制在7.5‰和9.5‰以下。
　　--0～6个月婴儿纯母乳喂养率达到50%以上；5岁以下儿童贫血患病率、生长迟缓率和低体重率分别控制在12%以下、7%以下和5%以下。
　　--新生儿访视率、7岁以下儿童健康管理率分别达到85%以上；国家免疫规划疫苗接种率以乡（镇）为单位达到90%以上。
　　--新生儿疾病筛查病种逐步扩大，新生儿先天性甲状腺功能减低症、苯丙酮尿症筛查率均达到90%以上，新生儿听力筛查率达到70%以上，免费孕前优生健康检查目标人群覆盖率达到80%以上，地中海贫血筛查率逐步提高，神经管缺陷发生率逐步下降。
　　--儿童艾滋病、梅毒、乙肝、结核病等重大传染病进一步得到控制；艾滋病感染孕产妇抗艾滋病毒用药率、所生婴儿抗艾滋病毒用药率均达到90%以上；梅毒感染孕产妇梅毒治疗率、所生儿童预防性治疗率均达到90%以上；乙肝感染孕产妇所生新生儿乙肝免疫球蛋白注射率达到95%以上。
　　--儿童肺炎、腹泻、贫血、哮喘、龋齿等常见病得到有效控制；肥胖、视力不良、儿童心理行为问题得到有效干预。

　　**三、**重点行动
　　（一）儿童健康促进行动
　　1.培养儿童健康生活方式。强化儿童养护人为儿童健康第一责任人理念，提高儿童养护人健康素养。以家庭、社区、托幼机构为重点，加大健康知识宣传力度，普及健康生活方式。
　　2.强化儿童健康管理。结合母子健康手册使用，扎实开展基本公共卫生服务项目中0～6岁儿童健康管理工作，为儿童提供全程医疗保健服务。促进儿童健康信息互联互通，逐步建立完善的儿童健康服务信息平台，推进儿童健康动态管理。
　　3.加强托幼机构卫生保健。加强托幼机构卫生保健业务指导和监督工作，促进儿童平衡膳食和适量运动，做好托幼机构突发公共卫生事件、传染病、常见病与多发病防控及食品安全、饮用水卫生、环境卫生等工作。
　　（二）新生儿安全行动
　　4.完善新生儿危重症救治网络。强化院内产儿科医生产前、产时及产后密切合作。提高院前急救机构反应能力，实现院前急救、院内急诊、重症监护有效衔接。建立省、市、县三级危重新生儿救治中心，健全分工负责、上下联动、应对有序、运转高效的救治、会诊、转诊网络。
　　5.加强新生儿保健和诊治。加强新生儿访视，指导家长做好新生儿喂养、护理和疾病预防，早期发现异常和疾病，及时处理和就诊。对早产儿进行专案管理，推动开展早产儿袋鼠式护理工作，改善早产儿生存质量。推广新生儿早期基本保健、新生儿复苏等适宜技术，提高新生儿保健工作水平。在贫困地区开展新生儿安全等项目，提高服务的可及性和公平性。
　　（三）出生缺陷综合防治行动
　　6.加强出生缺陷防治网络建设。建立覆盖城乡居民，涵盖婚前、孕前、孕期、新生儿各阶段的出生缺陷防治服务制度，强化“政府主导、部门协作、社会参与”的出生缺陷防治工作机制，完善出生缺陷防治相关标准和规范，健全服务网络，加强人才队伍建设，提高防治能力，推动出生缺陷防治工作规范、有序开展。
　　7.加强出生缺陷综合防治。加强科普宣传，倡导优生优育，强化群众出生缺陷风险防范意识。推动免费婚检和孕前优生健康检查城乡居民全覆盖，规范产前筛查和产前诊断工作，加强新生儿疾病筛查。加强对先天性心脏病、唐氏综合征、耳聋、地中海贫血等严重多发出生缺陷疾病的防治，减少先天残疾。完善出生缺陷患儿医疗保障制度，实施先天性结构畸形和遗传代谢病救助项目，减轻患儿家庭及社会负担。
　　（四）儿童早期发展行动
　　8.加强儿童早期发展内涵建设。建立符合儿童早期发展服务特点的产科、儿科（含新生儿科）、妇女保健科和儿童保健科等多学科协作机制，开展儿童早期发展专科建设。通过孕期营养、孕期心理、高危儿管理、儿童生长发育、儿童营养、儿童心理等方面的综合干预，充分开发儿童潜能，促进儿童体格、心理、认知、情感和社会适应能力的全面发展。
　　9.规范儿童早期发展服务。加强儿童早期发展示范基地建设，创新服务模式，规范机构管理，充分发挥基地引领带动作用，推进和规范儿童早期发展服务。开展儿童早期发展适宜技术培训，提高基层人员服务能力和技术水平。
　　10.促进儿童早期发展均等化。结合基本公共卫生服务，推动儿童早期发展均等化，在专业机构的指导下，促进儿童早期发展服务进农村、进社区、进家庭。实施母子健康发展综合项目，深入探索以农村为重点的儿童早期发展服务内容和服务模式。
　　（五）儿童营养改善行动
　　11.加强婴幼儿科学喂养。实施婴幼儿喂养策略，建立生命早期1000天营养咨询平台，强化医疗保健人员和儿童养护人婴幼儿科学喂养知识和技能。创新爱婴医院管理，营造爱婴爱母的良好社会氛围，保护、促进和支持母乳喂养。
　　12.改善贫困地区儿童营养。将贫困地区儿童营养改善项目作为提高国民素质的重要任务纳入健康扶贫工程整体推进，扩大项目覆盖范围，强化贫困地区儿童营养健康教育和辅食添加工作，提高营养包服用依从性，切实改善贫困地区儿童营养状况。
　　13.加强儿童肥胖监测和预防。开展儿童生长发育监测和评价，强化儿童个性化营养指导，引导儿童科学均衡饮食，加强体育锻炼，预防和减少儿童肥胖发生。实施儿童营养综合干预项目，研究开发儿童肥胖预防和干预适宜技术。
　　（六）儿童重点疾病防治行动
　　14.加强儿童传染病和常见病防治。实施国家免疫规划，开展儿童预防接种，加强艾滋病、梅毒、乙肝母婴阻断工作。以肺炎、腹泻、贫血、哮喘、龋齿、视力不良、心理行为问题等为重点，推广儿童疾病综合管理适宜技术，预防和减少儿童疾病发生。
　　15.开展儿童残疾防治。以视力、听力、肢体、智力残疾及孤独症为重点，加强0～6岁儿童残疾筛查。加强部门协作，推动0～6岁儿童残疾筛查、诊断、治疗、康复相衔接，使残疾儿童及时获得相关服务。
　　（七）儿童医疗卫生服务改善行动
　　16.推动儿童医疗卫生改革。完善儿童医疗卫生服务体系，扩大儿童医疗卫生服务资源，推进儿科医联体建设，鼓励社会力量举办儿童医疗保健机构。合理调整儿科医疗保健服务价格，推进分级诊疗制度，优先开展儿童家庭签约服务，增强基本公共卫生服务能力，逐步形成基层首诊、双向转诊的就医格局。加强儿童医疗保健人才队伍建设，加大培养和培训力度，提高薪酬待遇，促进职业发展。
　　17.改善儿童医疗保健服务。推进“互联网＋妇幼健康”，促进儿童健康服务信息化，开展儿童健康远程医疗服务。开展预约诊疗服务，实现分时预约，引导患者就近、错峰、有序就医。提供服务咨询、远程会诊、诊间结算、移动支付等服务，改善患儿及家长的就医体验。加强妇幼健康文化建设，为儿童提供温馨、舒适的就医环境。
　　18.发挥中医药在儿童医疗保健服务中的作用。加强医疗机构中医儿科建设，积极推广应用儿科中医适宜技术，推进儿童健康领域中医药公共卫生服务项目的实施。开展儿科中成药合理使用培训，提高医疗机构中医药防治儿童疾病能力。加强儿童重大疑难疾病中西医临床协作，提高儿科疑难病、急危重症诊疗水平。发挥中医治未病优势，开展中医药科普宣传及健康教育活动，推动中医药文化进家庭、进社区。
　　（八）儿童健康科技创新行动
　　19.加强儿童健康服务科技创新。在国家有关科技计划中，积极推进儿童健康领域项目的立项研究。聚焦儿科科技发展前沿和临床重大需求，重点围绕儿童重大疾病的预防、诊断、治疗、康复和健康管理，针对技术、方案和产品开展基础和应用研究。加强儿科科技创新基地平台建设，提升儿科临床研究能力。鼓励和支持研发生产儿童短缺药品、剂型和医疗器械。加强儿童健康政策研究，促进各级儿童健康工作决策水平和管理能力的提高。
　　20.推动成果转化和推广应用。发挥儿童健康相关机构和技术人员的医学创新主体作用，扩大机构和团队的创新成果使用和处置自主权，提高科研人员成果转化收益比例，促进儿童健康领域科技创新成果和适宜技术的推广和应用。

　　**四、**组织实施
　　（一）加强组织领导。各地要高度重视儿童健康工作，将其纳入健康中国建设和决胜全面建成小康社会的总体部署，结合实际制定本地区健康儿童行动计划和实施方案。加强督导评估，确保各项工作落到实处。
　　（二）保障经费投入。充分发挥各级政府的主体责任，加强对儿童健康服务网络、人才队伍建设、儿童健康服务的投入，加大对贫困地区的转移支付，提高服务的供给效率和公平性。
　　（三）广泛社会宣传。广泛宣传儿童健康方针政策，加强正面宣传、科学引导和典型报道，营造人人关心关注儿童健康、事事优先考虑儿童健康、爱婴爱母的良好社会氛围。
　　（四）开展国际交流与合作。实施中国全球卫生战略，充分展示我国儿童健康服务的成果和经验，积极参与儿童健康相关领域国际标准、规范的研究和制订，加强同“一带一路”建设沿线国家儿童健康领域的合作，推动联合国2030可持续发展目标的实现。

© 北大法宝：（[www.pkulaw.com](https://www.pkulaw.com/)) 专业提供法律信息、法学知识和法律软件领域各类解决方案。北大法宝为您提供丰富的参考资料，正式引用法规条文时请与标准文本核对。
欢迎查看所有[产品和服务](https://www.pkulaw.com/helps/2.html)。法宝快讯：[如何快速找到您需要的检索结果？](https://www.pkulaw.com/helps/70.html)[法宝](https://www.pkulaw.com/helps/69.html)V6有何新特色？

扫描二维码阅读原文

**原文链接：**<https://www.pkulaw.com/chl/9bf382e11b22ad0cbdfb.html>
